# To: Perfusion index for assessing microvascular reactivity in septic shock after fluid resuscitation

**DOI:** 10.5935/0103-507X.20190019

**Published:** 2019

**Authors:** Murat Daş, Okan Bardakci, Yavuz Beyazit

**Affiliations:** Department of Emergency Medicine, Canakkale Onsekiz Mart University - Canakkale, Turkey.; Department of Internal Medicine, Canakkale Onsekiz Mart University - Canakkale, Turkey.

**Dear Editor,**

In a recent issue of your journal, Menezes et al.^(1)^ presented a superb evaluation of the perfusion index in septic shock patients following fluid resuscitation. In a novel and fascinating piece of research, the authors seem to successfully evaluate microvascular reactivity byoximetry-derived perfusion index and reactive hyperemia. However, as already mentioned by the authors, there are a number of factors that can potentially impact on the results. Inlight of this, we would like to mention the important effect of patient position on perfusion index, which we believe warrants attention in evaluating the study's results.

Perfusion index derived from pulse oximetry provides a rapid indicator of microcirculatory changes and can help clinicians to detect abnormalities in the peripheral circulation.^(2,3)^ This method reflects real-time alterations in peripheral blood flow through use of a pulse oximeter device, which delivers readings noninvasively and continuously;^(4)^ however, it does have some flaws and limitations that should be accounted for during implementation. In this context, we believe that patient position is one of the important factors affecting the results of perfusion index measurement.

The effect of patient position on peripheral perfusion index is a novel topic; Tapar et al.^(5)^ were the first researchers to show that perfusion index values among healthy individuals vary according to body position, with use of supine measurements as a baseline. These authors demonstrated significant hemodynamic changes in different body positions, including supine, Trendelenburg, prone, and sitting supine. Moreover, in a study by Smith et al.,^(6)^ it was demonstrated that in conscious, healthy subjects, a head-up tilt triggers a prompt and balanced response, the immediate period being the first few seconds and the 'balanced' period being between 30 secs and 20 mins. This suggests that position alterations can cause blood pressure fluctuations, and that hemodynamic changes can cause alterations in peripheral perfusion index measurements.

In conclusion, we are of the opinion that in studies designed to measure the perfusion index in distinct disease states, the effect of the patient's position should be borne in mind.

*Murat Daş* *Department of Emergency Medicine,
Canakkale Onsekiz Mart University - Canakkale, Turkey.* *Okan Bardakci* *Department of Emergency Medicine,
Canakkale Onsekiz Mart University - Canakkale, Turkey.* *Yavuz Beyazit* *Department of Internal Medicine,
Canakkale Onsekiz Mart University - Canakkale, Turkey.*
